# Serum Vitamin D is Differentially Associated with Socioemotional Adjustment in Early School-Aged Ugandan Children According to Perinatal HIV Status and In Utero/Peripartum Antiretroviral Exposure History

**DOI:** 10.3390/nu11071570

**Published:** 2019-07-12

**Authors:** William Yakah, Jenifer I. Fenton, Alla Sikorskii, Sarah K. Zalwango, Robert Tuke, Philippa Musoke, Michael J. Boivin, Bruno Giordani, Amara E. Ezeamama

**Affiliations:** 1 Department of Food Science and Human Nutrition, Michigan State University, East Lansing, MI 48824, USA; 2 Department of Psychiatry, Michigan State University, East Lansing, MI 48824, USA; 3 Directorate of Public Health and Environment, Kampala Capital City Authority, Kampala 00256, Uganda; 4 Makerere University-Johns Hopkins University Research Collaboration, Kampala 00256, Uganda; 5 Department of Neurology and Ophthamology, College of Osteopathic Medicine, Michigan State University, East Lansing, MI 48824, USA; 6 Departments of Psychiatry, Neurology and Psychology, University of Michigan, Ann Arbor, MI 48109, USA

**Keywords:** vitamin D, antiretroviral therapy, perinatal HIV infection, Uganda, cognition, socioemotional adjustment

## Abstract

An impact of vitamin D in neurocognitive function has been theorized but it remains unknown whether vitamin-D insufficiency (VDI) is associated with worse socio-emotional adjustment (SEA) in vulnerable early school-aged children. This study examines the thesis that deficits in SEA are related to VDI using longitudinal data from 254 children that are perinatally HIV-infected (PHIV), exposed-uninfected (HEU), or unexposed-uninfected (HUU). In utero/peripartum antiretroviral (IPA) exposure was established per medical record documentation of biological mother’s ART regimen in pregnancy. Four caregiver-reported age- and sex-standardized measures of SEA were obtained at months 0, 6, and 12 for dependent children aged 6–10 years: externalizing problems (EPC), internalizing problems (IPC), behavioral symptoms index (BSI), and adaptive skills index (ASI). VDI was highly prevalent (74%, *n* = 188), and its association with change in SEA measures over 12 months varied by HIV-status (VDI*HIV, all *p*-values < 0.03). There was further variation in relationship of vitamin-D to SEA by IPA among PHIV (for ASI, BSI, and EPC, vitamin-D*IPA, *p*-value ≤ 0.01) and HEU (for BSI and EPC, vitamin-D*IPA, *p*-value ≤ 0.04). Among HUU, BSI (β = −0.32, 95% CI: −0.50, −0.13), IPC (β = −0.28, 95% CI: −0.47, −0.09), and EPC (β = −0.20, 95% CI: −0.37, −0.02) all declined moderately per quartile increment in VD. Among PHIV, on the one hand higher vitamin D predicted ASI gains (moderate vs. low VD, β = 0.52, *p* = 0.002), but this protective association was absent for BSI, EPC, and IPC (β = 0.36–0.77, *p* < 0.05). In absence of IPA-exposure, increasing vitamin-D predicted declines in BSI and EPC (moderate vs. low Vitamin D, β = −0.56 to −0.71, *p* ≤ 0.02) among HEU. However, given IPA exposure among HEU, higher VDI predicted moderate elevation in BSI (β = 0.39, 95% CI: 0.00, 0.78) and IPC (β = 0.48, 95% CI: 0.05, 0.92). Interaction between VD and IPA exposure for SEA outcomes among HEU and PHIV children warrants further investigation. The vitamin-D associated SEA improvement among HUU and HEU without IPA exposure suggests vitamin-D supplementation may remediate behavioral and adaptive deficits in this groups.

## 1. Introduction

Vitamin D insufficiency (VDI) and deficiency (VDD) are common in developed and developing countries, affecting more than 1 billion people worldwide [1,2]. VDI is associated with a range of socio-emotional deficits including depressive symptoms [3,4], endocrine dysregulation [5], anxiety [6], atypicality, and externalizing problems [7]. VD’s physiologic effect on health outcomes occurs through: (1) The genomic action of biologically active 1,25-(OH)2D on VD receptor (VDR)-a transcriptional factor and member of superfamily steroid nuclear receptor distributed in several body organs or (2) non-genomic action of VD, including rapid responses involving activation of Ca^++^ transport receptors in cell membranes [8,9]. VD also confers neurotrophic and neuroprotective benefits through regulation of glutamate-induced neurotoxicity [10]. Thus several physiologic processes—calcium metabolism, modulation of innate and adaptive immunity—are disrupted by VDD/VDI predisposing affected individuals to a range of morbidity [11].

Vitamin D is a neuro-steroid hormone with receptors in many specific brain structures like the cerebellum, hypothalamus, basal ganglia, thalamus, and hippocampus [12,13]. Of note is the expression of VDR in the hippocampus, part of the limbic system which significantly controls basic emotions and drives, learning and memory, neurogenesis and brain plasticity [14,15,16]. Therefore, maintaining optimal blood levels of vitamin D is crucial for maintaining calcium metabolism, blocking cellular proliferation/differentiation, immune modulation by promoting innate branch of immunity (activation of monocyte/macrophages) and inhibiting adaptive immunity by blocking IF-gamma, IL-1, and IL-2 [17].

Epidemiologic studies demonstrate that persons living with HIV/AIDS (PLWHA) are at higher risk of VDD/VDI relative to general population [18,19,20]. In Africa, a high prevalence of neuropsychiatric conditions, including social-emotional disorders, have increasingly been reported in this demographic [21]. With an increasingly recognized role of VD on a range of physiologic processes, the need to fully study the relationship between VDD/VDI and neurocognitive function among persons living with HIV/AIDS (PLWHA) has become critical. We have previously reported an association between VDD/VDI at HAART initiation and slower rate of CD4^+^ T-cell count recovery over 18 months in adult Ugandan PLWHA [22]. However, long term neurocognitive effects of VDI among HIV infected, or HIV-exposed uninfected children/adolescents remains poorly understood.

To inform existing knowledge gaps, this study investigates VDD/VDI-related deficits in SEA among Ugandan children that are perinatally HIV-infected (PHIV), HIV exposed uninfected (HEU) or HIV-unexposed uninfected (HUU). To our knowledge, no studies have investigated the association between low vitamin D and socio-emotional adjustment (SEA) in early school aged children in sub-Saharan Africa. We hypothesize that low vitamin D levels are associated with worse socio-emotional adjustment in early school-aged Ugandan children. We further hypothesize that this relationship may be modified by exposure to antiretroviral agents in both PHIV and HEU. An understanding of VDI/VDD, SEA, and ART relationships will inform the need for therapeutic interventions and clarify the need for appropriate targeting based on important modifiers.

## 2. Materials and Methods

### 2.1. Participants and Study Design

Participants were 6–10-year-old Ugandan children recruited with their adult primary caregivers between 15 March 2018, and 15 September 2018, as part of a larger year-long study of HIV and non-HIV related determinants of functional survival in HIV-affected children and community controls. Participants were enrolled from Kawaala Health Center (KHC)-a level 3 Health Centers that delivers the full range of antenatal care services in the Kawempe Division of Kampala, Uganda. This region has the highest prevalence of HIV (11%), compared to national prevalence of 7.3% [23]. By design, approximately equal numbers of children with perinatally acquired HIV infection (PHIV, *n* = 89), perinatally HIV exposed but uninfected (HEU, *n* = 84) and HIV unexposed uninfected (HUU, *n* = 83) community control children were enrolled. Of the 307 children in the parent study, laboratory assessment for vitamin D measures was implemented in 254 that form the study base for the current analysis. Current adult caregivers were enrolled regardless of their HIV status. PHIV were invited from children actively enrolled in HIV-care at KHC on a first come first serviced basis. As a birth cohort, HIV-exposed eligible children included in this study were largely born in the pre-antiretroviral therapy era. At the time of their birth, prevention of mother to child transmission of HIV was largely limited to intra-partum and neonatal single dose nevirapine [24] and few HIV-infected women received antiretroviral therapy for their own health. The following child demographic variables are objectively determined per medical records: prematurity (i.e., <37 weeks gestational age at birth), new born 5 min post-delivery wellbeing established per APGAR < 7 score, and low birthweight (≤2500 g). Long-term growth was established per height for age scores at enrolment.

### 2.2. Eligibility/Exclusion Criteria

Children were eligible for the study if born in a hospital/healthcare setting, were between 6 and 10 years old at enrolment, and had available health records from which objective data regarding general health condition—including the HIV status of biological mother, mode of birth, full or preterm birth, birthweight, participation in PMTCT services, any exposure to ART, type of ART exposure (if applicable), and HIV status of index child at birth or by date of discharge from the Early Infant Diagnosis program (if HEU) were established. Children born in non-clinic settings and child–caregiver pairs with missing antenatal register/delivery medical records were ineligible as key early life clinical variables for the child—such as prematurity, low birth weight, APGAR score, postnatal HIV status and the HIV status of biological mother in pregnancy could not be objectively established.

### 2.3. Statement of Ethical Approval

The study protocol was approved by the research ethics review committees of Michigan State University (IRB Protocol#: 16-828), Makerere University College of Health Sciences, School of Medicine (Protocol REC REF# 2017-017) and the Uganda National Council for Science and Technology (Protocol#: SS4378). All caregivers gave written informed consent and children provided assent for study participation.

### 2.4. Vitamin D Measurement

Venous blood was collected at time of enrolment, and serum fractions were separated and stored at −80 °C. Serum samples were shipped to the Sparrow Laboratories (East Lansing, MI, USA) for vitamin D analysis. Serum 25-hydroxyvitamin-D (25OHD), referred to as vitamin D in this paper) was measured by high performance liquid chromatography tandem mass spectrometry (HPLC-MS/MS) using the NHANES protocol as previously described [25]. Low, medium and high vitamin D status were respectively defined as serum 25-hydroxyvitamin-D levels of <20 ng/mL, 20–25 ng/mL and >25 ng/mL.

### 2.5. Measures of Socioemotional Adjustment

Socio-emotional adjustment (SEA) was measured using the Parent Rating Scales of the Behavioral Assessment System for Children (BASC-3) with assessments at enrollment, months 6 and 12. The BASC was adapted for cultural context, forward translated to Luganda and then back translated to Luganda as previously described [26]. Snacks were provided before each interview session to mitigate the distracting effect of hunger on responses. In the absence of local norms for BASC, SEA measures were internally age and sex standardized as follows: (raw-score-sample mean)/SD [26]. SEA quantifies a range of adaptive and problem behaviors that children may exhibit in home or community settings.

Four proxy reported composite SEA scales were defined based on caregiver response to 175 questions each rated on a four-point scale: never, sometimes, often and always. The composite scales include: externalizing problems composite (EPC), internalizing problems composite (IPC), behavioral symptoms index (BSI), and adaptive skills index (ASI). The EPC, IPC, and BSI quantify problematic behaviors and higher scores within these problematizing composites are indicative of worse SEA. On the other hand, higher scores in the ASI composite are indicative of higher SEA.

The EPC includes behaviors that are disruptive to both peers and adults such as hyperactivity, aggression, and conduct problems. The IPC consists of behaviors that do not typically manifest as disruptive but yet adversely affects peer relationship, including anxiety, depression, and somatization. The BSI estimates the general level of impairment within respondents and includes subscales: hyperactivity, aggression, depression, attention problems, atypicality and withdrawal. ASI summarizes behaviors reflective of appropriate emotional expression and control. These behaviors/characteristics are associated with successful functioning in home and school environments including: adaptability, activities of daily living, functional communication, social skills, and leadership. Composite scales have the advantage of summarizing performance and enabling investigators to reach broad conclusions of regarding the types of adaptive and maladaptive behaviors exhibited by children. Sub-scales on the other hand offer the advantage of specificity which is important for determining appropriate intervention approaches.

### 2.6. Other Key Covariates—Confounders and Effect Modifiers

#### 2.6.1. Early ART Exposure

As a birth cohort, HIV-exposed eligible children included in this study were born between 2008 and 2012, when implementation of ART strategies were expanding. Therefore, included in this cohort are some children whose HIV-positive mothers received no PMTCT intervention, some for whom prevention of mother to child transmission of HIV was limited to intra-partum and neonatal single dose nevirapine [27] and others for whom some version of ART intervention was offered. Intrapartum ART varied over time partly as a function of changes in standard of care and ART scale-up to include co-administration of AZT with or without 3TC and combination antiretroviral therapy for minority of HIV-positive pregnant women. Pregnant mothers who received ART were either on ART before pregnancy or were given intrapartum single dose nevirapine (sdNVP) ± AZT ± 3TC. All children received cotrimoxazole prophylaxis for prevention of bacterial infection.

#### 2.6.2. Perinatal HIV Status

Perinatal HIV status was established at birth and by 18 months of life via DNA PCR. Current HIV-status of HEU and HUU was objectively confirmed at enrolment via HIV-rapid diagnostic tests.

### 2.7. Statistical Analysis

Means and standard deviations (SD) for continuous variables and frequencies and percentages for categorical variables were calculated as part of descriptive measures to summarize child/caregiver sociodemographic and behavioral factors and baseline values of SEA. Summaries were made for the overall sample and by perinatal HIV status. Hypothesis testing for descriptive analyses were implemented using *t* tests for continuous variables and *Χ*^2^ tests for categorical variables.

Following descriptive univariate and bivariate analyses, multivariable repeated measures linear regression models were implemented to quantify associations between baseline vitamin D levels as primary determinant of change in SEA outcomes over 12 months follow-up using SAS PROC MIXED [28]. In all models, confounders—such as child’s age, sex, relationship with caregiver, caregivers’ age, sex, and socio-economic status, were adjusted for in light of subject matter knowledge. All analyses were performed with SAS version 9.3 (SAS Institute, Inc., Cary, NC, USA) and *p*-values of less than 0.05 were considered to be statistically significant in multivariable analyses. In addition, effect size (ES) estimates were calculated as β/√ (mean square error) of applicable multivariable models were derived for each comparison as a complimentary non p-value based measures of association to evaluate clinically meaningful differences. ES values are interpreted as follows: very small: ES < |0.20|, small: |0.20| ≤ ES < |0.30|, moderate: |0.30| ≤ ES < |0.50|, large: |0.50| ≤ ES < |0.80|, and very large: ES ≥ |0.80|.

## 3. Results

Child and caregiver sociodemographic are shown in Table 1. Per clinical thresholds for vitamin D levels regardless of HIV status, 47.6% of children were vitamin D deficient while 25.2% were vitamin D insufficient. However, due to the relatively low vitamin D levels in this population, vitamin D levels were re-classified as low (<20 ng/mL), medium (20–25 ng/mL), or high (>25 ng/mL). Serum vitamin D levels did not significantly differ by HIV status (*p* = 0.55). Additionally, unadjusted caregiver-reported indices of socioemotional adjustment did not differ by HIV status. The levels of vitamin D, regardless of definition, were associated with small and not statistically robust differences in SEA change in multivariable models adjusted for: time, child HIV status, APGAR score, height for age z-score and caregiver characteristics-age, sex, education, caregiving quality, depression, and alcohol use (Table 2). However, the relationship of vitamin D to change in SEA composite endpoints over 12 months according to child HIV status (HIV*VD, *p*-values ≤ 0.03).

In analyses evaluating vitamin D as an ordinal covariate with three levels (L, M, H), rising vitamin D levels was consistently associated with small to moderate reductions in behavioral symptoms, externalizing, and internalizing problems (β = −0.22 to −0.33, 95% CI: −0.51, −0.03) among community controls. These associations were confirmed and dose dependent in models evaluating vitamin D as categorical covariate. Specifically, relative to children with lowest vitamin D levels, high level vitamin D was associated with clinically large declines in behavioral symptoms and internalizing problems composite (β = −0.52 to −0.58, 95% CI: −2.70, −0.00) and clinically moderate decline in externalizing problems composite (β = −0.42, 95% CI: −0.86, 0.02). Among PHIV, there was no association between vitamin D levels – however defined and average change in BSI, EPC, and IPC over 12 months (Table 3).

Among HEU and PHIV, the relationship of vitamin D to change in several SEA composites varied in qualitatively (in terms of direction) and quantitatively (i.e., magnitude of association) by history of in utero, intrapartum, and peripartum exposure to anti-retroviral therapy (In utero ART * Vitamin D, *p*-value < 0.04 in 5 of 8 comparisons, Table 4 and Table 5).

On the one hand, among HEU without history of peripartum ART exposure, on average, increasing levels of baseline vitamin D predicted small to modesrate reductions in behavioral (β = −0.20; 95% CI: −0.45, 0.04), externalizing (β = −0.26; 95% CI: −0.49, −0.02), and internalizing problems (β = −0.13; 95% CI: −0.35, 0.29) over the study period. On the other hand, given any early life ART exposure among HEU, higher levels of vitamin D predicted an increase in behavioral symptoms (β = 0.47; 95% CI: 0.13, 0.81), externalizing (β = 0.32; 95% CI: −0.11, 0.74) and internalizing (β = 0.46; 95% CI: 0.10, 0.82) problems over the study period. However, the observed relationship between rising levels of vitamin D and respective SEA changes was not monotonic. Specifically, among HEU without history of early life ART, the associations between higher vitamin D and lower behavioral symptoms (β = −0.56; *p* = 0.02, ES = −0.54), internalizing (β = −0.40; *p* = 0.08, ES = −0.40), and externalizing (β = −0.71; *p* = 0.003, ES = −0.68) problems were strongest for comparison of children with medium vs. low vitamin D at baseline. Likewise, among HEU with any ART or sub-optimal ART exposure, higher externalizing problems and behavioral symptoms (β = 0.51 to 0.53; *p* = 0.09, ES = 0.49−0.51) were realized in relationship to medium vs. low vitamin D only. Although high vs. low vitamin D in relationship to respective SEA outcomes remained directionally consistent, associations were weaker (Table 4). 

Adjusted for current HAART regimen, rising vitamin D levels on the one hand predicted an increase in behavioral symptoms, internalizing and externalizing problems composites of SEA. On the other hand, increasing vitamin D levels predicted clinically moderate, though statistically insignificant, increases in adaptive skills particularly among PHIV without early ART exposure history and those exposed to sub-optimal ART in early life (medium vs. low vitamin D, β = 0.52, *p* = 0.002, ES = 0.64). With respect to problematizing SEA outcomes, highest vs. lowest vitamin D level at enrolment predicted moderate elevations in behavioral symptoms—internalizing and externalizing problems (β = 0.36 to 0.48, 95% CI: −0.51, −0.03) *among PHIV without early ART exposure history*. Likewise, baseline medium vs. low vitamin D predicted large elevations in behavioral symptoms—externalizing and internalizing problems SEA composites (β = 0.42 to 0.55; *p* = 0.26 to 0.085, ES = 0.47 to 0.66) *among PHIV with ART exposure history*. These associations were maintained in direction and strengthened in magnitude among an even smaller sub-group of PHIV whose early life ART exposure was sub-optimal (Table 5).

## 4. Discussion

In this sample of Ugandan early school-aged children with and without HIV-infection, more than 7 in 10 were vitamin D insufficient or deficient with low vitamin D levels in all groups regardless of perinatal HIV infection status. This high prevalence of vitamin D is in line with prior reports among children and adults in other countries, including in sub-Saharan Africa [20,29,30,31]. In line with our study hypothesis, we found that increasing vitamin D was associated with moderate decline in BSI, EPC, and IPC only among HUU children. This finding is consistent with higher vitamin D-related reduction in depressive symptoms among adolescent English children [32] and the previously reported relationship between low vitamin D levels and emotional problems/poor peer relationship among 3–17-year-old German children [33]. Our results are also in line with observed reductions in depressive symptoms over 3 months of vitamin D supplementation among Swedish adolescents with depression [34] and suggest the potential promise of vitamin D supplementation for enhancing socioemotional adjustment in the general population of vulnerable Ugandan children without HIV infection or exposure.

Although low vitamin D levels are associated with internalizing problems such as depression in HIV-uninfected populations [35], and a high prevalence of vitamin D insufficiency is commonly described in populations living with HIV [29], the relationship of low vitamin D to socio-emotional problems in HIV exposed children of HIV-positive women is unclear. Data from this study show that vitamin D related change in SEA indices over 12 months of follow-up in the entire cohort of PHIV are small. However, this relationship varied among PHIV and PHEU systematically according to presence/absence and type of peripartum ART exposure history. Among PHIV children, increasing vitamin D predicted an increase in behavioral symptoms and internalizing and externalizing problem composites of SEA regardless of peripartum ART exposure history. This adverse association between higher vitamin D level and SEA problems in PHIV was surprising and in contradiction with our study hypothesis.

**Figure 1:**
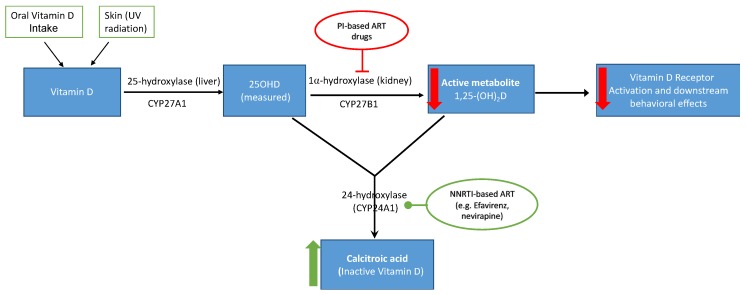
Proposed mechanism of how early ART exposure in utero/peripartum affects vitamin D metabolism in HIV infected and HIV exposed-uninfected children. Both PI-based and NNRTI-based ART regimen decrease availability of biologically active metabolite of vitamin D (1.25-(OH)D), leading to decreased to VDR activation and downstream effects.

Some studies have shown that ART decreases 25OHD in HIV patients [36,37] and some classes of ART drugs interact with vitamin D enzymatic pathway, reducing conversion of 25OHD to its biologically active form (i.e., 1,25-dihydroxyvitamin D, 1,25-(OH)_2_D) that acts on VD receptors (Figure 1). Of the three different ART class drugs, nucleoside reverse transcriptase inhibitors (NRTI), such as zidovudine (AZT) and lamivudine (3TC), are not expected to adversely affect vitamin D metabolism since they are not metabolized by cytochrome P450 enzymes [38]. However, non-nucleoside reverse transcriptase inhibitors (NNRTI) drugs such as efavirenz and nevirapine increase CYP24A1 activity and upregulate the conversion of 25OHD to its inactive metabolite 24,25-dihydroxyvitamin D rather than to 1,25(OH)_2_D (Figure 1) [39,40,41]. Lastly, protease inhibitors (PI), such as ritonavir, indinavir, and nelfinavir, decrease bioactivation of 25OHD by suppressing 1-alpha-hydroxylase, the critical enzyme for 1,25-(OH)D synthesis [42,43]. Among PHIV children in this study, 31% (*n* = 32) and 63% (*n* = 64), respectively, were currently on an NNRTI-and PI-based HAART for HIV-disease management. Therefore, ART exposure among PHIV children in this study possibly alters vitamin D metabolism, leading to insufficient levels of active 12,5-(OH)D necessary to bind to VDR and thus induce the associated physiologic and neurotrophic benefits.

Although rates of mother to child transmission (MTCT) have decreased, the number of HEU being born with peripartum ART exposure is rising exponentially [44]. The extent to which HEU neurodevelopmentally thrive in the long-term remains an active area of inquiry. In this study, increasing vitamin D levels were associated with SEA problems given a history of peripartum ART exposure among HEU. On the other hand, among HEU without peripartum ART exposure history, increasing vitamin D levels were associated with reductions in behavioral, internalizing, and externalizing problems as observed among HUU. Among HIV-exposed children with any peripartum exposure in this study, the vast majority were exposed to NRTIs, such as AZT and 3TC, which cross the placenta, interact with fetal DNA, and have the potential to induce genotoxicity and mitochondrial dysfunction in PHIV [45,46,47] and HEU, alike [48,49]. Mitochondrial toxicity also impairs the function of mitochondrial enzymes necessary for bioactivation of vitamin D, leading to systematic dysregulation of vitamin D metabolism in ART exposed children and the resultant higher VDD/VDI prevalence observed in these vulnerable groups.

Vitamin D status levels did not vary substantially by HIV status in this cohort but we speculate based on known biology of vitamin D metabolism that functional vitamin D deficiency was more common in HIV-exposed children as a function of peripartum ART exposure (as in HEU) and/or current HAART therapy (as in PHIV). The fact that vitamin-D related differences in SEA problems among HEU is counter-intuitive only among children with peri-partum ART exposure. This suggests that early ART-related alterations in vitamin D metabolism—whether through mitochondrial toxicity or adverse impact of nevirapine based HAART on vitamin D receptors are sustained through at least this period of development. These divergent associations of vitamin D for SEA outcomes have practical implication for vitamin D supplementation as a strategy for improving this neurocognitive disadvantage in HIV-exposed children. Some double-blind placebo-control studies have demonstrated that vitamin D supplementation can increase vitamin D levels in HIV-infected children and adults on ART [50,51], but whether this increase functionally correlates with higher active 1,25(OH)_2_D is unknown. It is also not clear that the relationship between vitamin D and SEA outcomes among HIV-exposed children with peripartum exposure to optimal cART will be the same as sub-optimal peripartum ART which was more common in this cohort.

The following additional study limitations lead to cautious interpretation of these results. First, we measured levels of 25OHD but not the biologically active form, 1,25-(OH)_2_D. Serum 25OHD levels are the most stable metabolite of vitamin D and circulating levels are a thousand fold higher than 1,25-(OH)_2_D [52]. Secondly, serum 25OHD levels were only measured at baseline. If vitamin D varied substantially over the study period, the impact of this variation would not be reflected in the described associations. Such an occurrence is more likely to result in under-estimation of the true association between vitamin D and SEA. Thirdly, in the absence of randomization to vitamin D in this study, residual confounding cannot be ruled out, despite adjustment for an extensive array of child and caregiver factors.

The emphasis on SEA in this study provides much needed insight into how well children are adjusting to their environmental contexts—an important distinction that has not been explored in previous studies. In addition to contributing data in understudied neurocognitive domains in this setting, a prospective cohort design with baseline assessment of vitamin D, three repeated assessments of SEA, and a rigorous analytic strategy would be key strengths that should enhance confidence in our observations. However, sub-group analyses within categories of early ART exposure are by definition preliminary and hypothesis generating. Of note, only 5 out of 84 HEU and 5 out of children 89 PHIV were exposed to optimal cART regimen in utero, suggesting results presented within these groups are extremely preliminary in nature and should be interpreted with an abundance of caution. Hence, future investigations in larger group of children are needed to confirm the observations described and test associated mechanisms.

## 5. Conclusions

In summary, we report hypothesis confirming association between vitamin D deficiency and worse socio-emotional deficits among HUUs and among HEU’s not exposed to early ART. We also report novel adverse associations between higher 25OHD and worse SEA problems among PHIV and HEU exposed to ART in the peripartum period. These counter intuitive associations may reflect the well described iatrogenic dysregulation of vitamin D metabolism due to anti-retroviral therapy in HIV-infected or early ART in HEU. While vitamin D supplementation may be a possible intervention target to remediate SEA impairments among HUU and HEU without peripartum ART exposure, the interaction between VD and early ART for neurocognitive outcomes in children with early ART exposure or current ART requires further elucidation and confirmation. Hence, future mechanistic studies of interactions between vitamin D, various ART regimen, and alterations in vitamin D receptor function have the potential to inform therapeutic strategies to rescue any ART-related interruption in vitamin D metabolic pathway and thus enhance physiologic impact of vitamin D supplementation in peripartum ART exposed children—if warranted.

## Figures and Tables

**Table 1:** Socio-demographic and clinical description of perinatally exposed, uninfected, or infected children born to HIV-infected/uninfected women from Uganda at 6–10 years of life.

	Overall (*n* = 254)	PHIV (*n* = 89)	HEU (*n* = 84)	HUU (*n* = 83)	*p*-Value ^ɤ^
**Child Sociodemographic and Clinical Characteristics**					
Vitamin D (ng/mL)	21.2 ± 7.1	21.7 ± 6.7	21.2 ± 7.2	20.6 ± 7.4	0.55
Low Vitamin D, *n* (%)	130 (51.2)	44 (49.4)	41 (48.8)	44 (53.0)	
Medium Vitamin D, *n* (%)	63 (24.8)	23 (25.8)	21 (25.0)	19 (22.9)	
High Vitamin D, *n* (%)	61 (24.0)	21 (23.6)	21 (25.0)	19 (22.9)	
Female Child *n* (%)	123 (48.4)	41 (46.1)	46 (54.8)	38 (45.8)	
Child Age (in years, mean ± SD)	7.7 ± 1.4	7.9 ± 1.5	7.5 ± 1.4	7.6 ± 1.4	0.31
Height for age *z*-score (mean ± SD)	−0.25 ± 1.1	−0.62 ± 0.9	−0.14 ± 1.1	0.06 ± 1.0	<0.001
Apgar Score (5 min post-delivery)	7.6 ± 2.5	7.8 ± 2.2	7.6 ± 2.5	7.5 ± 2.7	0.41
**Peripartum Antiretroviral therapy Exposure History**					
No early life ART Exposure	192 (45.6)	60 (68.8)	50 (59.5)	82 (100)	<0.001
sdNVP ± AZT ± 3TC	52 (20.47)	23 (26.1)	29 (34.5)	0 (0)	
Combination ART	10 (3.9)	5 (5.7)	5 (6.0)	0 (0)	
**Caregiver Socio-demographics**					
Female caregiver, *n* (%)	236 (92.9)	83 (93.3)	78 (92.8)	77 (92.8)	0.99
Married or living with a sexual partner	102 (46.6)				
Caregiver has own income	183 (72.0)	65 (73.0)	61 (72.6)	59 (71.1)	0.99
Caregiver Depressed, *n* (%)	86 (33.9)	22 (27.4)	39 (46.4)	27 (32.5)	0.005
Caregiver age (years, mean ± SD)	34.8 ± 8.5	34.5 ± 8.7	36.6 ± 8.4	33.4 ± 8.3	0.05
Highest CG Quality (5th Quintile)	43 (16.9)	17 (19.1)	16 (19.0)	12 (14.4)	0.68
Caregiver Reported Cognitive Scores *					
Adaptive Skills Index (ASI)	−0.01 ± 1.0	0.03 ± 0.9	−0.06 ± 1.0	−0.01 ± 1.1	0.79
Behavioral Symptoms Index (BSI)	−0.03 ± 1.0	−0.1 ± 0.9	0.05 ± 1.0	−0.03 ± 1.0	0.60
Internalizing Problems Composite (IPC))	0.02 ± 1.0	0.02 ± 1.0	0.05 ± 1.0	−0.02 ± 1.0	0.84
Externalizing Problems Composite (EPC)	−0.02 ± 1.0	−0.08 ± 0.9	0.08 ± 1.1	−0.05 ± 0.9	0.80

* ASI includes child proficiency in the following five areas: adaptability, social skills, leadership, activities of daily living and functional communication. BSI: captures extent of child having the following problematic behaviors: attention problems, atypicality, withdrawal, depression, hyperactivity, aggression; IPC includes display of behaviors consistent with: depression, anxiety, somatization; EPC: captures children’s display of following problematic behaviors: hyperactivity, aggression, conduct problems. ɤ: *p*-value estimates difference in proportion (via chi-square tests) or difference in means (via *t*-tests) for children by HIV status. PHIV: perinatally HIV-infected; HEU: HIV exposed uninfected; HUU: HIV-unexposed uninfected.

**Table 2:** The relationship of vitamin D to socio-emotional adjustment indices among early school-aged Ugandan children varies by HIV Status **.

	Perinatally HIV Infected	Perinatally HIV Exposed Uninfected	HIV Unexposed Uninfected	HIV Status × Vitamin D interaction
	Adjusted LSM ± SE	Adjusted LSM ± SE	Adjusted LSM ± SE	*p*-Value
ASI				0.03
Low Vitamin D	−0.09 ± 0.14	0.08 ± 0.16	0.12 ± 0.17	
Medium Vitamin D	0.37 ± 0.16	−0.13 ± 0.19	0.01 ± 0.20	
High Vitamin D	0.10 ± 0.17	0.20 ± 0.18	0.14 ± 0.21	
Partition Analysis **	F-value (*p*-value)	F-value (*p*-value)	F-value (*p*-value)	
F-test (2, 224)	5.03 (0.007)	1.14 (0.32)	0.19 (0.83)	
BSI				0.04
Low Vitamin D	−0.13 ± 0.18	0.14 ± 0.18	0.12 ± 0.20	
Medium Vitamin D	0.06 ± 0.20	0.02 ± 0.22	−0.17 ± 0.26	
High Vitamin D	0.03 ± 0.18	0.35 ± 0.22	−0.46 ± 0.23	
Partition Analysis **	F-value (*p*-value)	F-value (*p*-value)	F-value (*p*-value)	
F-test (2, 224)	0.64 (0.53)	0.72 (0.49)	3.70 (0.03)	
EPC				0.11
Low Vitamin D	−0.01 ± 0.17	0.27 ± 0.19	0.09 ± 0.22	
Medium Vitamin D	0.15 ± 0.17	−0.01 ± 0.22	−0.03 ± 0.27	
High Vitamin D	0.06 ± 0.17	0.58 ± 0.29	−0.32 ± 0.24	
Partition Analysis **	F-value (*p*-value)	F-value (*p*-value)	F-value (*p*-value)	
F-test (2, 224)	0.38 (0.68)	2.03 (0.13)	1.76 (0.17)	
IPC				0.05
Low Vitamin D	0.01 ± 0.16	0.02 ± 0.16	0.17 ± 0.19	
Medium Vitamin D	0.08 ± 0.19	−0.03 ± 0.19	−0.40 ± 0.21	
High Vitamin D	0.15 ± 0.18	0.11 ± 0.19	−0.31 ± 0.20	
Partition Analysis **	F-value (*p*-value)	F-value (*p*-value)	F-value (*p*-value)	
F-test (2, 224)	0.37 (0.69)	0.21 (0.81)	4.47 (0.01)	

Estimates shown are age and sex standardized difference in mean socio-emotional adjustment indicator in relationship to vitamin D levels. ASI = adaptive skills index, BSI = Behavioral Symptoms Index, EPC = externalizing problems composite, IPC =internalizing problems composite. Calculated from repeated measures linear mixed models adjusted for: time, child (APGAR, HAZ), and caregiver (parent vs. non-parent status, age, education, caregiving quality, caregiver depression, current alcohol use). ** Partition analysis for global test of OHD level related difference in SEA measure within HIV-groups. Household ID is specified as random effect, subject effect = child ID, covariance matrix.

**Table 3:** Baseline Vitamin D level related average change in socio-emotional adjustment indices within strata of perinatal HIV infection.

Outcome	Exposure	PHIV		HEU		HUU		HIV × Vitamin D Interaction
		Difference (95% CI)	ES	Difference (95% CI)	ES	Difference (95% CI)	ES	*p*-Value
Adaptive Skills Index	Vitamin D Level							**0.04**
	Per Unit increment	0.14 (−0.02, 0.32)	0.15	0.03 (−0.17, 0.21)	0.03	0.00(−0.17, 0.21)	0.00	
	Low	Ref	Ref	Ref	Ref	Ref	Ref	
	Medium	**0.47 (0.18, 0.77)**	**0.55**	−0.21 (−0.60, 0.17)	−0.25	−0.11 (−0.54, 0.31)	−0.13	
	High	0.19 (−0.16, 0.55)	0.22	0.11 (−0.28, 0.51)	0.13	0.02 (−0.39, 0.43)	0.02	
Behavioral Symptoms Index	Vitamin D Level							**0.04**
	Per Unit increment	0.10 (−0.08, 0.27)	0.11	0.11 (−0.14, 0.35)	0.12	**−0.29 (−0.50, −0.08)**	**−0.31**	
	Low	Ref	Ref	Ref	Ref	Ref	Ref	
	Medium	0.20 (−0.19, 0.58)	0.21	−0.09 (−0.51, 0.34)	−0.09	−0.11 (−0.54, 0.31)	−0.12	
	High	0.16 (−0.19, 0.52)	0.17	0.25 (−0.27, 0.76)	0.27	0.02 (−0.39, 0.43)	0.02	
Externalizing Problems Composite	Vitamin D Level							0.11
	Per Unit increment	0.06 (−0.13, 0.24)	0.07	0.12 (−0.16, 0.40)	0.14	−0.20 (−0.39, 0.02)	−0.23	
	Low	Ref	Ref	Ref	Ref	Ref	Ref	
	Medium	0.17 (−0.21, 0.56)	0.19	−0.28 (−0.71, 0.15)	**−0.32**	−0.12 (−0.62, 0.37)	−0.14	
	High	0.08 (−0.27, 0.43)	0.09	0.31 (−0.27, 0.90)	**0.35**	−0.42 (−0.86, 0.02)	**−0.47**	
Internalizing Problems Composite	Vitamin D Level							0.05
	Per Unit increment	0.11(−0.08, 0.30)	0.12	0.20(−0.07, 0.47)	0.22	**−0.28 (−0.51, −0.06)**	**−0.31**	
	Low	Ref	Ref	Ref	Ref	Ref	Ref	
	Medium	0.12 (−0.26, 0.49)	0.13	−0.05 (−0.42, 0.31)	−0.06	**−0.49 (−0.93, −0.04)**	**−0.55**	
	High	0.20 (−0.18, 0.58)	0.22	0.45 (−0.12, 1.03)	**0.51**	**−0.52 (−0.97, −0.08)**	**−0.59**	

Estimates shown are age and sex standardized difference in mean socio-emotional adjustment indicator in relationship to vitamin D levels. Calculated from repeated measures linear mixed models adjusted for: time, child (Apgar, HAZ), and caregiver (age, sex, education, caregiving quality, caregiver depression, current alcohol use) factors in all strata. ASI = adaptive skills index, BSI = Behavioral Symptoms Index, EPC = externalizing problems composite, IPC = internalizing problems composite. Among HEU, the model is further adjusted for history of maternal ARVs. Among PHIV, model is additionally adjusted for maternal ARV + current ART regimen. ES measures are provided to compliment difference of means. Bolding indicates at least moderate clinical or statistical significance (*p* < 0.05).

**Table 4:** Baseline Vitamin D level related average change in socio-emotional adjustment within strata of early ART exposure type among HEUs.

Outcome	Exposure	Among HEU without Early ART Exposure (*N* = 50) *		Among HEU with Any early ART Exposure (*N* = 34) **		Among HEU with Sub-Optimal ART Exposure (*N* = 29) ***		VD × Early ART
		LSM ± SE	β (*p*-Value), ES	LSM ± SE	β (*p*-Value), ES	LSM ± SE	β (*p*-Value), ES	*p*-Value
Adaptive Skills Index	Vitamin D Level							0.31
	Low	0.31 ± 0.13	Ref	−0.10 ±0.18	Ref	−0.22± 0.19	Ref	
	Medium	−0.06 ± 0.19	**−0.37 (0.11), −0.42**	0.01 ± 0.24	0.11 (0.71), 0.13	0.01 ± 0.24	0.22 (0.48), 0.25	
	High	0.05 ± 0.28	−0.25 (0.42), −0.29	0.17 ± 0.15	**0.27 (0.24), 0.31**	0.14 ± 0.18	**0.36 (0.18), 0.42**	
Behavioral Symptoms Index	Vitamin D Level							0.04
	Low	0.52 ± 0.18	Ref	0.58 ± 0.18	Ref	0.62± 0.20	Ref	
	Medium	−0.03 ± 0.15	**−0.56 (0.02), −0.54**	1.10 ± 0.26	**0.53 (0.09), 0.51**	1.16 ± 0.26	**0.54 (0.10), 0.52**	
	High	0.50 ± 0.18	−0.03 (0.93), −0.03	0.72 ± 0.25	0.14 (0.64), 0.14	0.95± 0.24	**0.32 (0.30), 0.31**	
Externalizing Problems Composite	Vitamin D Level							0.01
	Low	0.62± 0.18	Ref	0.63± 0.21	Ref	0.65 ± 0.19	Ref	
	Medium	−0.08± 0.15	**−0.71 (0.003), −0.68**	1.14 ± 0.22	**0.51 (0.09), 0.49**	1.19 ± 0.23	**0.53 (0.07), 0.54**	
	High	0.55 ± 0.27	−0.10 (0.83), −0.11	0.81± 0.25	0.18 (0.59), 0.17	1.01± 0.25	**0.36 (0.27), 0.35**	
Internalizing Problems Composite	Vitamin D Level							0.25
	Low	0.37± 0.15	Ref	0.43± 0.17	Ref	0.55± 0.19	Ref	
	Medium	−0.03 ± 0.16	**−0.40 (0.08), −0.40**	0.84 ± 0.22	**0.41 (0.12), 0.41**	0.88 ± 0.21	**0.32 (0.24), 0.32**	
	High	0.54 ± 0.27	0.18 (0.53), 0.18	0.77± 0.27	**0.33 (0.30), 0.33**	0.91± 0.29	**0.36 (0.30), 0.36**	

Estimates shown are age and sex standardized difference in mean socio-emotional adjustment indicator in relationship to vitamin D levels. Calculated from repeated measures linear mixed models adjusted for: time, vitamin D, caregiver depression, caregiving quality. Effect size interpreted according to the following rubric: very small: ES < |0.20|, small: |0.20| ≤ ES < |0.30|, moderate: |0.30| ≤ ES < |0.50|, large: |0.50| ≤ ES < |0.80|, and very large: ES ≥ |0.80|. This means that if two groups’ means do not differ by 0.2 standard deviations or more, the difference is trivial, even if it is statistically significant. ES measures are provided to compliment difference of means. *p*-value corresponds to hypothesis test for consistency in vitamin D related changes within stratum of early ART exposure type. * Among HEU without early ART exposure (*n* = 50), there were 23, 17, and 10 respectively were classified as low, medium, and high vitamin D. ** Among HEU with any early ART exposure (*n* = 34), 16, 6, and 12 were respectively classified as low, medium and high vitamin D. ***: Among HEU with sub-optimal ART exposure (*n* = 29), 13, 6, and 10 were respectively classified as low, medium and high vitamin D. This sample excludes 5 HEU perinatally exposed to optimal combination ART. Bolding indicates statistical significance (*p* < 0.05) or at least moderate clinical significance.

**Table 5:** Baseline Vitamin D level related average change in socio-emotional adjustment within strata of early ART exposure type among perinatally HIV infected children.

Outcome	Exposure	Among PHIV without Early ART Exposure (*n* = 60)		Among PHIV with Any Early Exposure Any ART (*n* = 28)		Among PHIV with Sub-optimal Early ART Exposure (*n* = 23)		VD × Early ART
		LSM ± SE	β (*p*-Value), ES	LSM ± SE	β (*p*-Value), ES	LSM ± SE	β (*p*-Value), ES	*p*-Value **
Adaptive Skills Index	Vitamin D Level							0.01
	Low	−0.16 ± 0.13	Ref	0.09 ± 0.15	Ref	−0.02 ± 0.16	Ref	
	Medium	−0.36 ± 0.14	**0.52 (0.002), 0.64**	0.34 ± 0.30	0.26 (0.12), 0.32	0.37 ± 0.28	0.39 (0.20), 0.48	
	High	−0.02 ± 0.15	0.14 (0.49), 0.17	0.26 ± 0.22	0.18 (0.59), 0.22	0.39 ± 0.32	**0.42 (0.25), 0.52**	
Behavioral Symptoms Index	Vitamin D Level							<0.001
	Low	−0.15 ± 0.13	Ref	−0.13 ± 0.20	Ref	−0.01 ± 0.21	Ref	
	Medium	−0.16 ± 0.17	0.00 (0.99), 0.00	0.36 ± 0.35	**0.49 (0.19), 0.54**	0.65 ± 0.36	**0.66 (0.09), 0.73**	
	High	0.29 ± 0.16	**0.45 (0.02), 0.50**	−0.32 ± 0.18	−0.19 (0.39), −0.21	−0.45 ± 0.19	−0.44 (0.08), −0.48	
Externalizing Problems Composite	Vitamin D Level							0.002
	Low	−0.10 ± 0.11	Ref	0.01 ± 0.14	Ref	0.10 ± 0.14	Ref	
	Medium	−0.04 ± 0.17	0.05 (0.75), 0.06	0.56 ± 0.30	**0.55 (0.09), 0.66**	0.86 ± 0.29	**0.77 (0.01), 0.93**	
	High	0.26 ± 0.14	**0.36 (0.0495), 0.43**	−0.16 ± 0.15	−0.17 (0.36), −0.21	−0.23 ± 0.17	−0.33 (0.13), −0.40	
Internalizing Problems Composite	Vitamin D Level							0.13
	Low	−0.06 ± 0.14	Ref	−0.04 ± 0.21	Ref	0.07 ± 0.29	Ref	
	Medium	−0.08 ± 0.17	−0.02(0.89), −0.02	0.44 ± 0.33	**0.42 (0.26), 0.47**	0.57 ± 0.36	**0.50 (0.22), 0.56**	
	High	0.42 ± 0.18	**0.48 (0.01), 0.54**	−0.03 ± 0.26	−0.06 (0.84), −0.07	−0.09 ± 0.24	−0.16 (0.62), −0.18	

Estimates shown are age and sex standardized difference in mean socio-emotional adjustment indicator in relationship to vitamin D levels. Calculated from repeated measures linear mixed models adjusted for: time, current ART regimen vitamin D, caregiver depression, caregiving quality. Effect size interpreted according to the following rubric: very small: ES < |0.20|, small: |0.20| ≤ ES < |0.30|, moderate: |0.30| ≤ ES < |0.50|, large: |0.50| ≤ ES < |0.80| and very large: ES ≥ |0.80|. This means that if two groups’ means do not differ by 0.2 standard deviations or more, the difference is trivial, even if it is statistically significant. ES measures are provided to compliment difference of means. **: *p* -value corresponds to hypothesis test for consistency in vitamin D related changes within stratum of early ART exposure type. Among PHIV without early ART Exposure (*n* = 60), there were 31, 16, and 13 respectively were classified as low, medium and high vitamin D. Among PHIV with Sub-optimal ART Exposure (*n* = 23), 10, 6, and 7 were respectively classified as low, medium and high vitamin D. Among PHIV with any early ART exposure (*n* = 28), 12, 8, and 8 were respectively classified as low, medium and high vitamin D. Bolding indicates statistical significance (*p* < 0.05) or at least moderate clinical significance.
